# Endoscopic Repair of a Full Thickness Hypopharyngeal Defect Using Acellular Dermal Matrix

**DOI:** 10.1177/01455613231206293

**Published:** 2023-10-16

**Authors:** Benjamin T. Ostrander, Frederic J. Kolb, Philip A. Weissbrod

**Affiliations:** 1 Department of Otolaryngology, Head and Neck Surgery, University of California San Diego, La Jolla, CA, USA; 2 Division of Plastic Surgery, Department of Surgery, University of California San Diego, La Jolla, CA, USA

## Clinic

Endoscopic repair of pharyngeal and hypopharyngeal defects is a technically difficult procedure with limited application due to challenges with adequate tissue availability and mobilization for closure. Most defects require open surgical approaches and utilize soft tissue flap reconstruction to achieve adequate closure.^1–3^ These methods often involve complex procedures, large donor sites, prolonged healing times, and high complication rates.

Dermal substitutes, such as ADM, may enable endoscopic repair, circumventing more invasive approaches. These biologics have several advantages, including that they can be used over poorly vascularized tissue or bone lacking periosteum, and do not require autologous donor tissue harvest.^3,4^ Once such dermal substitute is Integra^®^, an ADM comprised of a silicone layer on top of a porous matrix of bovine collagen and shark-derived chondroitin-6-sulfate glycosaminoglycan.^4^

Several recent reports have demonstrated success in using ADMs to augment reconstruction after resection of hypopharyngeal cancer.^1,5^ However, there are no reports detailing the use of ADMs for use in endoscopic repair of hypopharyngeal defects.

A 72-year-old female with a history notable for remote hypopharyngeal cancer status postradiation 20 years prior presented with a posterior hypopharyngeal defect. She recently underwent an endoscopic gastroduodenoscopy during treatment for ovarian cancer. She then developed dysphonia, dysphagia, and odynophagia, found to be due to a hypopharyngeal perforation. She was made nil per os and referred to our group for further management due to a nonhealing wound with exposed spine in the hypopharynx.

**Figure 1.**
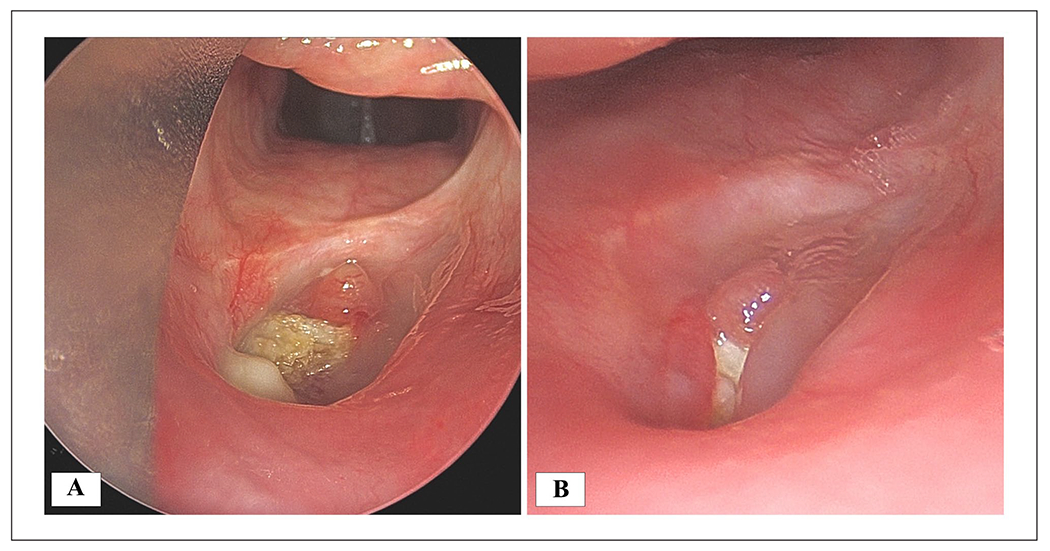
(A) Initial direct laryngoscopy 8 months after injury demonstrated necrotic full thickness hypopharyngeal defect in previously irradiated tissue. (B) A persistent hypopharyngeal defect was noted on direct laryngoscopy 12 months post-injury.

Direct microlaryngoscopy with biopsy 8 months after injury revealed a 1.5 cm × 1.5 cm ulcerative lesion in the midline posterior hypopharynx with necrotic-appearing bone, consistent with osteoradionecrosis and iatrogenic injury (Figure 1A). Pathology revealed squamous mucosa with ulceration and no malignancy. This lesion was followed for an additional year and did not heal.

**Figure 2.**
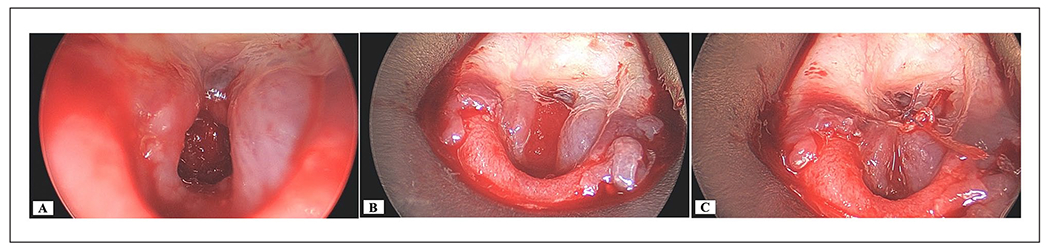
Intraoperative photographs demonstrating technique for endoscopic closure with ADM. (A) The hypopharyngeal wound was debrided down to healthy bone. (B) A sheet of Integra^®^ ADM was cut to 12 mm × 9 mm, the silicone layer was removed, and inserted under the mucosal margins and over the exposed bone. (C) The graft was secured using endoscopic suturing. Vicryl 4.0 on a TF needle was used to secure the graft and approximate the wound edges. A second Vicryl 6.0 on a S29 needle was used to close the inferior aspect of the defect.

**Figure 3.**
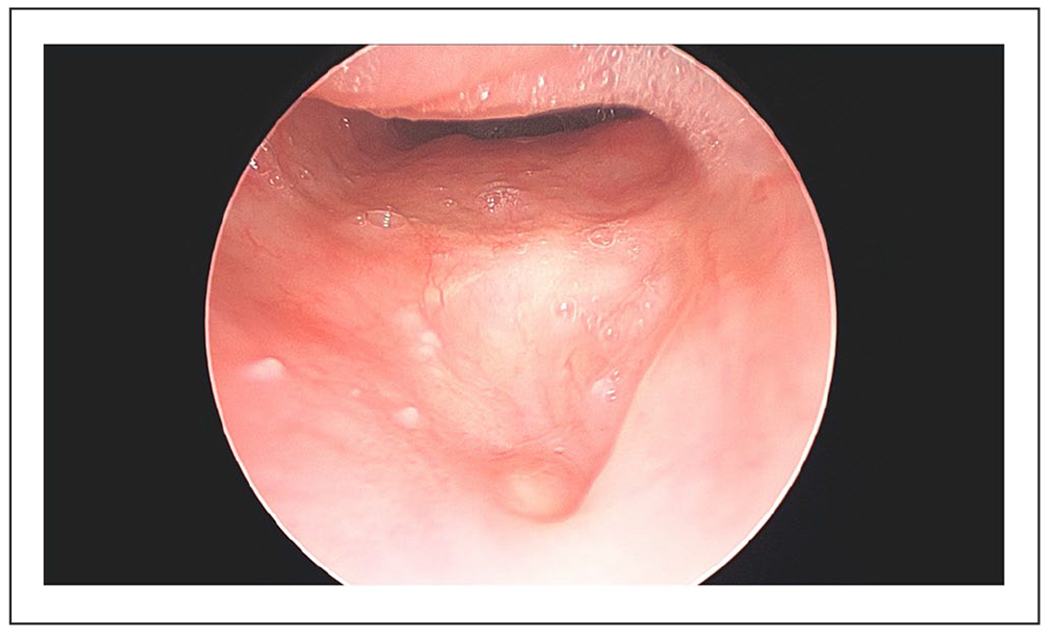
Repeated direct microlaryngoscopy 10 weeks after repair with acellular dermal matrix revealed a well-healed, fully mucosalized hypopharyngeal defect.

She was scheduled for definitive repair with plan for a regional or free flap reconstruction. At this time, examination revealed partial resolution of the mucosal defect, which was now 4 mm × 3 mm in size (Figure 1B). Given the reduced size, we opted to attempt endoscopic closure and avoid open surgical repair. The bone was debrided with cups forceps until healthy bleeding tissue was exposed (Figure 2A). A flap elevator was used to elevate the mucosal margins in all directions and the edges were freshened sharply. The wound was now 8 mm × 6 mm. Powdered vancomycin was applied to the defect and a sheet of Integra^®^ was cut to 12 mm × 9 mm, the silicone layer was removed, and inserted under the mucosal margins and over the exposed bone (Figure 2B). Vicryl 4.0 on a Tetralogy of Fallot (TF) needle was used to secure the graft and approximate the wound edges. A second Vicryl 6.0 on a S29 needle was used to close the inferior aspect of the defect (Figure 2C). Fibrin glue was applied over the repair. The patient was admitted and discharged on postoperative day 1, with a 4-week course of intravenous antibiotics to cover for osteomyelitis. Repeated direct microlaryngoscopy 10 weeks later revealed a well-healed, fully mucosalized hypopharyngeal defect (Figure 3). She started to eat by mouth again, with mild residual dysphagia related to postradiation changes.

In summary, we report the first case of endoscopic repair of a full thickness hypopharyngeal defect using ADM. By waiting several months for the mucosa to partially granulate by secondary intention, the hypopharyngeal defect decreased to a size that the authors felt comfortable attempting endosopic repair with ADM. In doing so, a transoral endoscopic approach could be used, avoiding external incisions, donor site morbidity, and longer recovery from a more extensive approach. This approach is particularly useful for irradiated hypopharyngeal mucosal defects, which have lower propensity to spontaneously heal.

Despite use as a supplement to flap reconstruction, there have been no published examples of ADMs being utilized in endoscopic hypopharyngeal repair. Endoscopic repair of wounds, in general, is technically difficult and limited by challenges of advancing tissue sufficiently to accomplish tension-free closure. The addition of ADM as an option potentially allows for larger wounds to be addressed endoscopically. Posterior midline hypopharyngeal wounds lend themselves nicely to this concept as the graft can be wedged in the retropharyngeal space between the spine and pharynx and secured in place with endoscopic suturing. We were able to place a pair of Vicryl sutures through the edges of the pharyngeal defect and graft to prevent migration. For a larger wound, ADM could be sutured circumferentially to the mucosal edges. The authors believe a full thickness defect of approximately 2 cm^2^ could be addressed using this method. This case demonstrates the feasibility of this approach for covering hypopharyngeal defects using an endoscopic approach.

## Significance Statement

Most pharyngeal and hypopharyngeal defects require open surgical approaches and utilize flap reconstruction to achieve closure. Endoscopic repair of these defects is technically challenging, with limitations due to problems with adequate tissue availability and mobilization. We present the first report of endoscopic repair of a nonhealing full thickness hypopharyngeal defect using acellular dermal matrix (ADM), with durable closure and improved swallowing function, demonstrating the feasibility of an endoscopic technique for challenging nonhealing defects.
